# AliquotG: An Improved Heuristic Algorithm for Genome Aliquoting

**DOI:** 10.1371/journal.pone.0064279

**Published:** 2013-05-14

**Authors:** Zelin Chen, Shengfeng Huang, Yuxin Li, Anlong Xu

**Affiliations:** State Key Laboratory of Biocontrol, Guangdong Key Laboratory of Pharmaceutical Functional Genes, College of Life Sciences, Sun Yat-sen University, Guangzhou, Guangdong, People’s Republic of China; University of Lausanne, Switzerland

## Abstract

An extant genome can be the descendant of an ancient polyploid genome. The genome aliquoting problem is to reconstruct the latter from the former such that the rearrangement distance (i.e., the number of genome rearrangements necessary to transform the former into the latter) is minimal. Though several heuristic algorithms have been published, here, we sought improved algorithms for the problem with respect to the double cut and join (DCJ) distance. The new algorithm makes use of partial and contracted partial graphs, and locally minimizes the distance. Our test results with simulation data indicate that it reliably recovers gene order of the ancestral polyploid genome even when the ancestor is ancient. We also compared the performance of our method with an earlier method using simulation data sets and found that our algorithm has higher accuracy. It is known that vertebrates had undergone two rounds of whole-genome duplication (2R-WGD) during early vertebrate evolution. We used the new algorithm to calculate the DCJ distance between three modern vertebrate genomes and their 2R-WGD ancestor and found that the rearrangement rate might have slowed down significantly since the 2R-WGD. The software AliquotG implementing the algorithm is available as an open-source package from our website (http://mosas.sysu.edu.cn/genome/download_softwares.php).

## Introduction

Whole genome sequencing projects permit easy and accurate detection of genome rearrangement events by direct comparison of two genome sequences. To measure these events, Sankoff proposed the use of the edit distance in 1992, which is defined as the minimum number of rearrangement events necessary to transform one genome into another. Several types of edit distance have been proposed, including the reversal distance and the double cut and join (DCJ) distance [1]. Pevzner *et al*. introduced reversal distance and developed a breakpoint graph–based, linear–time exact algorithm for computation [2]–[5]. Later, Yancopoulos *et al.* proposed the DCJ distance and its corresponding efficient calculation [6]. DCJ differs from other edit distances in that it includes chromosomal fusion, fission, inversion, translocation and block interchange within a single model and allows simpler algorithms for calculation.

In many species, such as in vertebrates [7], [8], paramecia [9], yeasts [10] and many plants [11], the extant rearranged genome descended from an ancient form that underwent *r*–way polyploidization (or whole genome duplication, WGD, where *r*–way indicates that polyploidization generated *r* copies of the original genome). For example, two almost consecutive rounds of WGD (2R–WGD), or 4–way polyploidization, occurred upon the origin of vertebrates and are suggested to be responsible for the dramatic increase in the morphological complexity of vertebrates [12]. Some researchers have suggested that WGDs might increase rearrangement rates [13]–[17], whereas others have proposed the opposite [18]. Despite the debate regarding whether polyploidization complicates the rearrangement process, polyploidization has posed challenges for the computation of rearrangement distance. One of the challenges is the genome aliquoting problem [19], which is to find the minimum number of rearrangement events required to transform a rearranged *r*–way polyploidized genome into a non–rearranged form and reconstruct the genome of the latter. The problem with *r* = 2 represents a special case called genome halving, which has been addressed several times, and for which a linear–time exact algorithm with respect to DCJ distance is available [20]–[23]. The more generalized case of the problem with *r* >2 has also been studied [19], [24], [25], including the heuristic algorithm developed by Warren and Sankoff [19]. Their algorithm relies on the occurrence number of gene–gene adjacencies in the extant genome, which is an effective characteristic when the rearrangement distance is small and many gene–gene adjacencies retain more than two copies. However, in reality, many gene–gene adjacencies could appear only once and hence affect the precision of the algorithm.

In this study, we introduce a new heuristic algorithm for the genome aliquoting problem with respect to DCJ distance. Our algorithm considers not only the multiplicities of edges in the CPG but also the number of black cycles and their local costs (i.e., N_p_ and C_p_ in Methods), thus it can handle weak adjacencies. This algorithm performs well on extensive simulation data sets and is better than previous methods. It has been successfully applied to the 2R-WGD of vertebrates.

## Methods

### Problem Description

Warren and Sankoff have introduced the genome aliquoting problem [19]. Here, we revisit the problem and present the definitions and notations that are commonly used.

**Figure 1:**
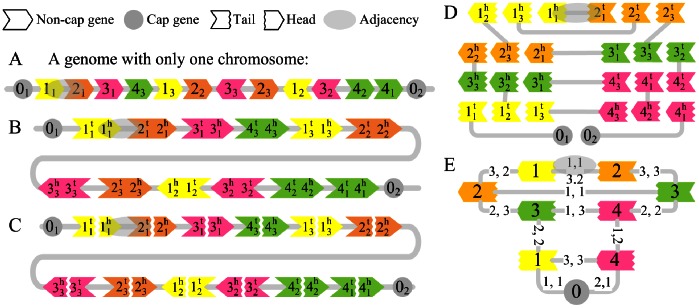
Representation of a genome, a PG and a CPG. (A) A rearranged duplicated genome with duplicated size of 3 is represented as a sequence of signed integers, where a positive (negative) sign is represented by the direction of the colored arrow. (B) The same genome is represented as a sequence of extremities (i.e., heads, tails or cap genes). (C) PG of the above genome. Each non-cap gene is cut into head and tail, which becomes two vertices in the partial graph. (D) The vertex reposition of the PG in (C). (E) The contracted PG that is converted from the PG showed in (C) and (D). Each vertex corresponds to an extremity family. Numbers on each edge indicate the copy IDs (i.e., subscripts) of the two extremities connected by the corresponding adjacency. Note that the edge (1^h^, 2^t^) in (E) corresponds to 2 adjacencies (or edges) in (C), so its multiplicity is 2.

The fundamental element in a genome is the gene, and several duplicated (or similar) genes form a gene family. The number of genes in a gene family represents the size of the family. In this paper, a gene family is represented by an unsigned integer that is called a (gene) family ID, while a gene is represented by its signed family ID with an integer subscript, where the sign (positive or negative) indicates direction of the gene in the genome, while the subscript, named copy ID, denotes different genes in the same family. We represent a genome as a set of linear chromosomes, in which each chromosome is represented by a sequence of genes (Figure 1A) beginning and ending with two special genes called *cap genes*. All cap genes form the *cap family* with family ID ‘0’. We can also represent a genome by replacing each non-cap gene +g_i_ with an ordered pair, 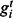 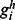, and by replacing −g_i_ with 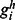 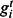 (Figure 1B), where 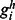 and 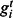 are called the *head* (ID) and *tail* (ID) of the gene g_i,_ respectively, and both heads and tails (and the cap genes) are called *extremity* (ID). The heads or tails of genes within the same gene family also consist of a *head family* or *tail family*, both of which (along with the cap family) are called the *extremity family* (with the ID g^t^ and g^h^, for example). The connection between two adjacent genes is defined as an *adjacency*. Moreover, we define three special genomes: (1) *single copy genome*, in which the size of each non-cap gene family is exactly one; (2) *perfectly duplicated genome*, which represents multiple copies of the single copy genome, and may descend from a single copy genome through WGD events; (3) *rearranged duplicated genome*, in which all non-cap gene families have exactly the same size, named *duplicated size*.

To describe the genome aliquoting problem, a rearrangement operation (or model) is needed. Here we use the DCJ operation [6]. Each DCJ operation cuts two adjacencies and rejoins the four free extremities differently to form two new adjacencies. If both of the original adjacencies connect a non-cap gene and a cap gene, and the two cap genes are from different chromosomes, the operation fuses the two chromosomes; and if one adjacency connects two cap genes and the other connects two non-cap genes, the operation breaks a chromosome into two. The DCJ distance between two genomes is defined as the minimum number of DCJ operations necessary to transform one genome into the other [6].

Now, the *genome aliquoting problem* is defined as follows. Given a rearranged duplicated genome (G_obs_, 'obs' stands for observed) with duplicated size *r* (≥2), reconstruct a perfectly duplicated genome (G_dup_) such that the DCJ distance between them is minimal [19]. Additionally, we can also obtain the corresponding single copy genome (G_anc_) of G_dup_. It remains an open question whether an efficient exact solution exists for the genome aliquoting problem with respect to DCJ distance [19]. Here, we present a new heuristic algorithm to solve the problem.

### Solutions

Our solution makes use of the *partial graph* (PG). A PG of a genome is a graph in which each vertex corresponds to an extremity of the genome, and each edge corresponds to an adjacency (Figure 1C). All of the above concepts regarding extremities are also suitable for vertices, e.g., the subscript of vertex is also called the ‘copyID’ of the vertex. PGs of two genomes with same gene contents can form a bicolor-edge graph (i.e., breakpoint graph [21]), one color for each genome, which is used to calculate the DCJ distance between the two genomes. Minimizing DCJ distance is the same as maximizing the number of alternating (color) cycles in the bicolor graph [6]. A PG can be transformed into a new graph through the following two steps: (1) contract all vertices of the same extremity family into a single new vertex, identified by the corresponding family ID, and (2) each original edge, taking 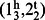 for example, is transformed into a new edge (1^h^, 2^t^) with multiplicity 1. In this latter step, if the edge already exists then the multiplicity of that edge is increased by 1 every time. Any edge with multiplicity 0 is removed. The new graph is called *contracted partial graph* (CPG). Figure panels 1C to 1E show an example of the process transforming a PG into a CPG.

We define an edge in CPG(G_obs_) (i.e., the CPG of G_obs_) strong if its multiplicity is greater than one, otherwise the edge is called weak. The corresponding adjacency of a strong (weak) edge is also called strong (weak) adjacency. Because every edge in CPG(G_dup_) must have multiplicity *r* (i.e., the duplicated size of G_obs_), a strong edge in CPG(G_obs_) with higher multiplicity (>1) represents the true original edge in CPG(G_dup_) more likely than weak edges. We initialize graph A as CPG(G_obs_) and graph B as PG(G_obs_) (i.e., the PG of G_obs_), with all edges colored black. The solution iterates the following first two steps and then goes to Step 3.

### Step 1: Infer Strong Adjacencies

Taking multiplicity as edge weight by applying the maximum weight-matching algorithm to all strong edges of graph A, we first obtain a matching, which contains a set of pairwise non-adjacent strong edges. For each edge (u, v) in the matching, we add a gray edge (u, v) with the multiplicity *r* (i.e., the duplicated size of G_obs_) into graph A.

Secondly, for each (strong) edge (u, v) in the matching, we add *r* pairwise non-adjacent gray edges (u_i_, v_j_) into graph B. To know which u*_i_* (*i = *1, •••, *r*) and v*_j_* (*j = *1, •••, *r*) should be paired, we use the following local greedy method. We search for a set of paths from u to v in graph A with minimum total length (in terms of number of edges) in all path sets that satisfy the following four conditions: (1) each path in the set must start and end with black edges; (2) each path must visit and leave a matched vertex (i.e., vertex in the matching) through two edges with different colors; (3) an edge cannot be passed more times than its multiplicity; (4) each unmatched vertex (i.e., vertex not in the matching) can be visited at most once by at most one path in the set. This routine is declared as **find_path**(u, v) and is implemented using a modified Suurballe’s algorithm [26].

**Figure 2:**
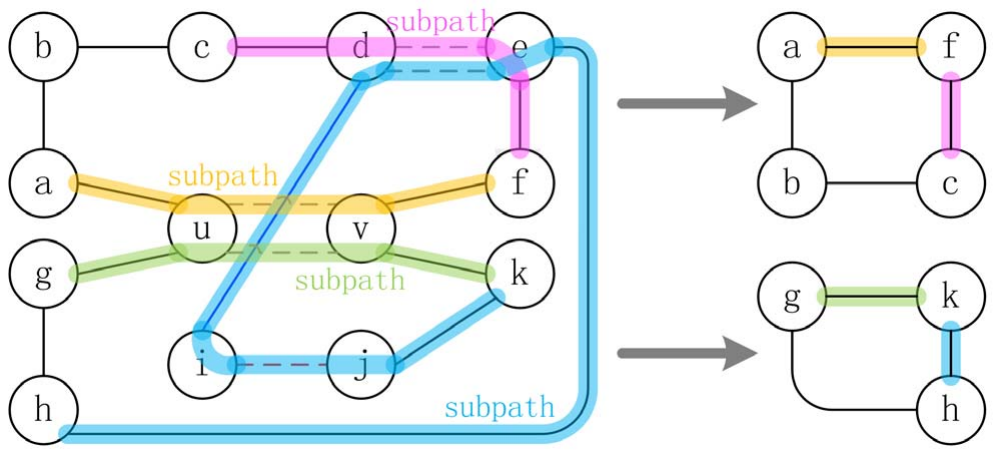
Transforming paths to black cycles. Vertices u, v are matched. The upper path is (u, a, b, c, d, e, f, v), and the bottom path is (u, g, h, e, d, i, j, k, v). Four colored subpaths on the left are contracted into four edges in the right cycles, respectively. An odd path is transformed into an even cycle, while an even path is transformed into an odd cycle. Solid and dashed edges correspond to black and gray edges, respectively.

Each path in the set and the gray edge (u, v) will form a cycle with a length that is the length of the path plus one, so an odd path will form an even cycle and vice versa. Because of the path conditions (1) and (2), any gray edge in the path is contained in some alternating black-gray subpath whose first and last edges are black, therefore each subpath (e.g., (c, d, e, f) in Figure 2) can be contracted into a single black edge (e.g., (c, f) in Figure 2). Therefore, all gray edges are removed, and all edges in the cycle will be black after being contracted (Figure 2). Condition (4) guarantees that all the black cycles are separated. According to Theorems 4 and 8 in Alekseyev’s and Pevzner's paper [20], to maximize the number of alternating cycles in graph A and B, all odd black cycles (i.e., even paths in the path set, 'odd' or 'even' in terms of the number of edges) should be paired.

**Figure 3:**
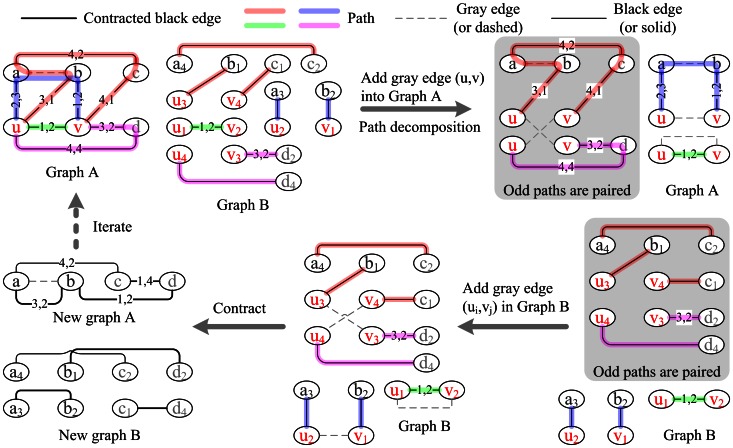
Adjacency inference in H (i.e., gray dashed edges in graph B) from matched vertices u, v in graph A. Vertices a, b are matched vertices that have not been contracted. Vertices c, d are unmatched. Black solid edges are derived from PG(G_obs_), CPG(G_obs_) or contracted edges. The four different paths found by **find_path(u, v)** are as follows: (1) (u, v) (green), (2) (u, a, b, v) (blue), (3) (u, b, a, c, v) (orange), and (4) (u, d, v) (pink). In graph A, the first two paths are odd and form two even cycles – (u, v, u) and (u, a, b, v, u) – by adding the gray edge (u, v) in the top right panel. The former disappears after contraction, while the latter generates a new black edge (a, b) in the bottom left panel. The last two paths are merged by two same gray edges (u, v) to form an even cycle – (u, b, a, c, v, u, d, v, u) – that is contracted and generates two new black edges, (b, d) and (c, d). Two numbers on each edge of graph A indicate the copy IDs of the two corresponding vertices in graph B.

Therefore, for each odd path, let (u, a,•••, b, v) be the path in graph A in which (u, a) and (b, v) are black (by condition (1)), and let (u_i_, a_x_), (b_y_, v_j_) be the corresponding edges in graph B. We add a gray edge (u_i_, v_j_) into graph B (Figure 3, paths 1 and 2) and decrease the multiplicity of the edge (u, v) by one in graph A. For each pair of even paths (u, a,•••,b, v) and (u, c,•••,d, v), let (u_i_, a_x_), (v_j_, b_y_), (u_p_, c_w_), (v_q_, d_z_) be the corresponding edges in graph B of (u, a), (v, b), (u, c), (v, d), then we add two gray edges (u_i_, v_q_), (u_p_, v_j_) into graph B (Figure 3. paths 3 and 4) and decrease the multiplicity of edge (u, v) in graph A by two. For any other copy of edge (u, v) in graph A, do as in the former case. Next, we remove u, v and all the edges incident to them from graph A. Now, for each gray edge (u_i_, v_j_), both u_i_ and v_i_ are incident to exactly one black edge in graph B (Figure 3, bottom middle panel). As an example, consider the gray edge (u_2_, v_1_) in Figure 3. There is one black edge (a_3_, u_2_) incident to u_2_ and another black edge (v_1_, b_2_) incident to v_1_. These three edges (two black edges and one gray edge) are contracted into a new black edge (a_3_, b_2_), and the multiplicity of the edge (a, b) is increased by a factor of one in graph A (Figure 3, bottom left panel, thick edge). The new graph B is contracted into a new (contracted partial) graph A. At last, we add all adjacencies (u_i_, v_j_) into genome H (which is empty initially), corresponding to the above gray edges (u_i_, v_j_) in graph B.

### Step 2: Infer Weak Adjacencies

**Figure 4:**
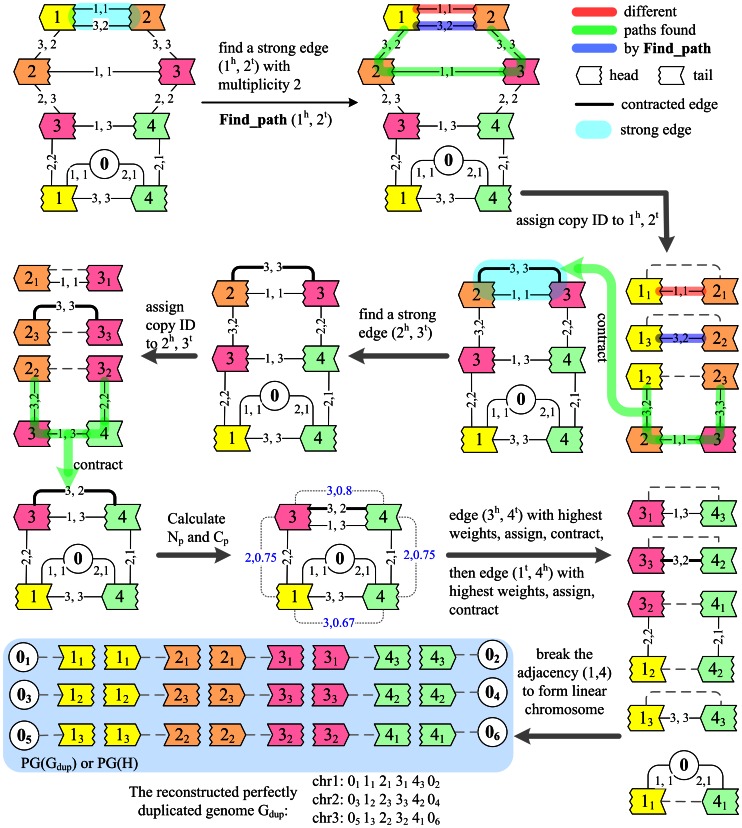
Reconstructing G_dup_ from the genome (G_obs_) of **Figure 1A**. This figure shows how graph A changes during the solution. In this simple example, step 2 is not necessary, but here we still calculate the weights N_p_, C_p_ and use step 2 to infer the last two adjacencies 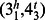, 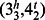, 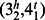, 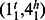, 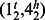 and 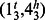 of G_dup_ (or H) ahead of linearizing circular chromosomes, to show how step 2 works. Different vertex colors indicate different gene families. Edges with multiplicity *k* are represented as *k* edges for a clear display. The numbers on each edge are the copy IDs of the two vertices incident to the corresponding edge in graph B. Blue numbers are the weights N_p_, C_p_ for the pair of vertices linked by gray dotted edge.

At this stage, all vertices in the matching and all gray edges in graph A have been contracted into black edges, so only unmatched vertices and black edges remain present in the new graphs A and B. If graphs A and B are empty, the iteration finishes, otherwise, we attach each pair of vertices u, v in graph A two new weights (e.g., Figure 4): (1) the number of paths, denoted as N_p_, that **find_path(u, v)** returns, which indicates how many black cycles can be generated – according to Theorems 4 and 8 in Alekseyev and Pevzner's paper [20] and the statement in Step 1, the more black cycles, the larger the number of alternating cycles in the breakpoint graph of G_obs_ and G_dup_; (2) the average number of alternating cycles that can be yielded from the black cycles per edge cost, denoted as C_p_, which is calculated as follows: C_p_ = 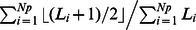, where *L_i_* is the length of the *i*th path in the path set. We choose the pair of vertices u, v with the highest combined weight W_p_ = *a*N_p_+(1−*a*)C_p_ (*a* = 0.5), match u with v, and then form new adjacencies of H as in Step 1. All matched non-cap vertices are removed from graphs A and B after contracting, so each non-cap vertex is matched only once. Therefore, the genome H will be a perfectly duplicated genome at last.

In the case of the genome halving problem, graph A in Step 2 can be decomposed into black cycles easily. For each pair of vertices u, v in a black cycle of graph A, N_p = _2. Let L be the length of the black cycle, then the total length of the two paths found by **find_path** is 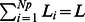 always. However, there are three cases with a different C_p_: (1) when *L* is even and the u, v vertices are separated by an odd number of edges, the two paths are both odd, so 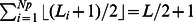, and C_p_ = (*L*+2)/2*L*; (2) when *L* is even and u, v are separated by even number of edges. The two paths are both even, so 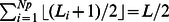 and C_p_ = L/(2L) = 0.5; (3) when *L* is odd, one path – L_1_, for example – is even, while the other – L_2_ for example – is odd, so 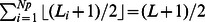 and C_p_ = (L+1)/2L. Because (L+2)/2L>(L+1)/2L >0.5, vertices u, v in case (1) are chosen for even black cycles and u, v in case (3) are chosen for odd black cycles. Therefore, if we follow the above process, PG(H) (PG of H) will be non-crossing (defined in [20]), and the maximum number of alternating cycles in PG(G, H) (PG of G and H, i.e., breakpoint graph of G, H) will be the same as in Theorem 7 of [20]. Though the above solution is exact for the genome halving problem, it can only be a heuristic principle for the genome aliquoting problem with duplicated size larger than two, where the black edges in graph A cannot be decomposed into black cycles easily.

To speed up the process, we do not calculate W_p_ in an all–to–all way, but only calculate it for those pair of vertices for which the distance in graph A is not larger than a given value **Depth** (which is set to one in the simulation).

### Step 3: Linearize Circular Chromosomes

After the above iteration, genome H becomes the reconstructed and perfectly duplicated genome G_dup_. However, we do not restrict the genome to be linear throughout, so we have to address all circular chromosomes. First, we try to break any circular chromosome and merge it into another chromosome if the DCJ distance between G_obs_ and the new genome G_dup_ is not larger than that between G_obs_ and the old G_dup_. This approach is just a DCJ operation between an adjacency in the circular chromosome and an adjacency in another chromosome. Alternatively, we try to search for an adjacency in the circular chromosome on the same principle and break it to form a new linear chromosome. If both searches fail, we search for a DCJ operation to merge or break the circular chromosome with the least increase of DCJ distance. At last, we calculate the DCJ distance between the reconstructed G_dup_ and G_obs_ using the method of Yancopoulos *et al.* ([6]). A simple example of this method is shown in Figure 4.

## Results

### Simulation Results

To evaluate the performance of our heuristic algorithm, we created four simulation datasets, each with 1,000 data points. Each data point is generated as follows: an integer sequence 1,···,*n* is generated and randomly broken into *m* segments to simulate an ancestral single copy genome just before WGD (G_anc_) with *m* chromosomes and *n* gene families. Variable *m* is uniformly randomly chosen between 1 and *m*_0_ (*m*_0_ = 2 if *n* = 100 and *m*_0_ = 10 if *n* = 1,000). To simulate an *r*–way WGD, G_anc_ is further duplicated into *r* copies, and genes from the same gene family are assigned different copy IDs, from 1 to *r*, to obtain the perfectly duplicated genome (G_dup_) with the duplicated size *r*. Once G_dup_ is generated, *D*_N_ DCJ operations are performed to rearrange G_dup_ into the extant rearranged duplicated genome (G_obs_), where *D*_N_ is uniformly distributed in the interval [1, *r*×*n*]. Given any DCJ distance *D*_DCJ_ between G_obs_ and G_dup_, the corresponding relative DCJ distance (*d*_DCJ_) is defined as *D*_DCJ_/(*r*×*n*) (where *r*×*n* is just the number of genes in G_obs_). At last, a complete data point contains three simulated genomes (G_anc_, G_dup_ and G_obs_), and both G_dup_ and G_obs_ consist of *n* gene families with a size of *r*.

**Figure 5:**
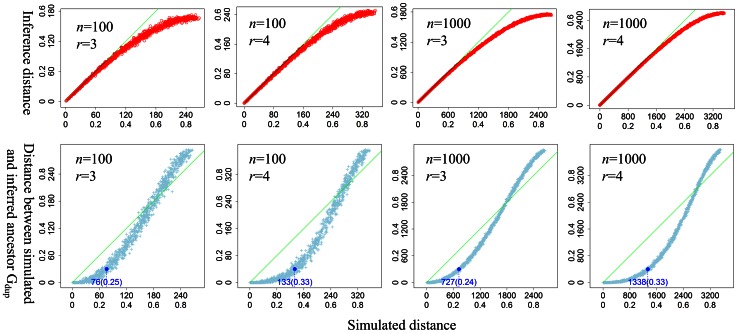
Application of the heuristic algorithm to four simulation datasets. Each light blue point in each panel corresponds to a data point. Green line: x = y. Inner axis labels represent DCJ distance, whereas outer labels show relative DCJ distance. The Y value of the dark blue dot is 0.1 (relative DCJ distance) in each plot, where *n* is the number of gene families and *r* is the duplicated size of G_obs_, and the blue numbers (DCJ distance and relative DCJ distance) below each blue cycle represent the corresponding X values. Note that the inferred and simulated distances are almost the same when the relative simulated distance is smaller than 0.4. The distance is less than 0.1, until the relative simulated distance increases to approximately 0.6. The relative DCJ distance between simulated and inferred G_dup_ is small when the simulated distance is smaller than 0.25 (*r* = 3) or 0.33 (*r* = 4).

For each data point, we applied our heuristic algorithm to infer G_dup_ from G_obs_ and computed DCJ distance between them. The reconstructed G_dup_ is called inferred G_dup_, and the DCJ distance is called *inferred DCJ distance* (or inferred distance), accordingly. The DCJ distance between the simulated G_dup_ and G_obs_ is called *simulated DCJ distance* (or simulated distance). Comparing the two distances shows that the inferred distance is almost the same as the simulated distance when the relative simulated distance is lower than 0.4 (Figure 5, top). Only a few data points (<1%) have an inferred distance larger than the simulated distance by 1–2 DCJ operations. When the relative simulated distance is larger than 0.4, the algorithm displays significant underestimation of the simulated distance (Figure 5, top). It is the parsimony in the definition of the genome aliquoting problem that leads to this underestimation; furthermore, in practice, the smaller the inferred distance, the more accurate the algorithm.

Another important application of this algorithm is to infer the ancestral duplicated genome G_dup_. Therefore, we also compared the simulated G_dup_ with the inferred G_dup_ using the distance between them (Figure 5, bottom). We found that the distance between simulated G_dup_ and inferred G_dup_ was almost 0 when the relative simulated distance was smaller than 0.1 for *r* = 3, and 0.2 for *r* = 4, which means that the inferred G_dup_ is almost the same as the simulated G_dup_. The distance remains far smaller than the simulated distance as the relative simulated distance increases to 0.25 for *r* = 3 or to 0.33 for *r* = 4, suggesting that at medium divergence our algorithm displays good accuracy in the ancestral genome reconstruction. However, when the relative simulated distance rises over 0.4 for *r* = 3 or over 0.6 for *r* = 4, the inferred G_dup_ is nowhere near the simulated G_dup_.

**Figure 6:**
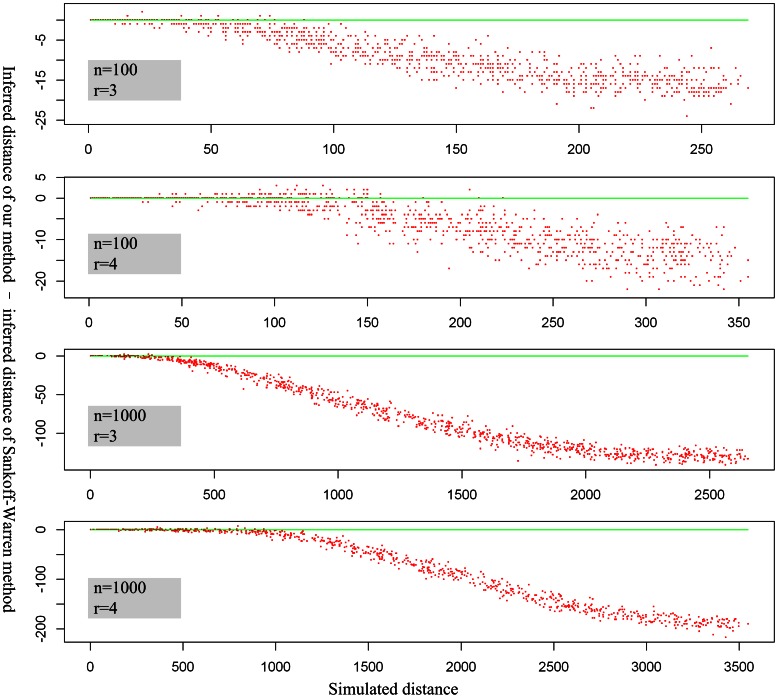
Comparison of inferred distance computed using Sankoff-Warren’s algorithm and our own algorithm. Each red point is a simulation data point. The proportions of data points calculated using our method with inferred distances larger than those calculated using the Sankoff-Warren method are 0.7%, 5.9%, 0.5% and 3.2% (from the upper to bottom panels, respectively). Green line: y = 0. The variable *n* is the number of gene families, and *r* is the duplicated size of G_obs_.

Moreover, we compared our algorithm with that of Sankoff and Warren [19]. We used Sankoff-Warren’s algorithm to reconstruct G_dup_ from the simulated G_obs_ of the same simulation datasets and compared the inferred DCJ distance between the two algorithms. The definition of the genome aliquoting problem suggests that the smaller the inferred distance the better the method. We found that the distance inferred by our algorithm was smaller than that inferred by Sankoff-Warren’s algorithm in more than 95% of cases, especially when the relative simulated distance exceeded 0.25 (i.e., there were more weak adjacencies in G_obs_), which suggests that our method is more accurate, especially as the simulated distance increases (Figure 6). Further checking the result suggests that our inferred distance is always smaller than that obtained by Sankoff-Warren’s algorithm when we do not break circular chromosomes.

Taken together, although our heuristic algorithm cannot guarantee minimum DCJ distance between G_obs_ and the reconstructed G_dup_, the simulation results shows that it usually performs well, even when the relative simulated distance exceeds 0.4.

### Application to the 2R-WGD Event of Vertebrates

It has been recognized that two rounds of WGD (2R–WGD) happened at the origin of vertebrates 450 Mya [7]. Because the interval between the two rounds of WGD is supposed to be short, the 2R–WGD can be assumed to be a 4–way polyploidization. Consistent with this, most phylogenetic relationships between ohnologs cannot be reliably resolved. Moreover, it was suggested that few rearrangement events had occurred during the 2R–WGD [27]. To analyze the 2R–WGD, we applied our algorithm to human, mouse and chicken genomes.

The gene repertoire and corresponding genomic coordinates of human, mouse and chicken were download from ENSEMBL FTP (ftp://ftp.ensembl.org/pub/release-50/fasta/) [28]. The complete gene repertoire of amphioxus *Branchiostoma foridae* was downloaded from JGI (http://genome.jgi-psf.org/Braﬂ1/Braﬂ1.download.ftp.html, the file ’proteins.Braﬂ1.fasta.gz’ and ’Bﬂoridae_v1.0_FilteredModelsMappedToAssemblyv2.0.gff.gz’). Because amphioxus is a close relative to vertebrates and did not undergo the 2R–WGD, amphioxus genes can be used as anchors to identify vertebrate gene families that were created by the 2R–WGD. For vertebrate–vertebrate pairs, only 1∶1 orthologous gene families from OMA [29] were retained for further study. We implemented Dehal's method [7] to infer amphioxus–vertebrate 1∶4 2R-WGD-paralogs, using amphioxus as an outgroup. The numbers of genes used for study are presented in Table 1.

**Table 1:** The number of genes used in the analysis.

	amphioxus	chicken	mouse
chicken	168	–	–
mouse	284	9,983	–
human	328	9,729	14,058

We found that the inferred relative DCJ distances from the three genomes were in agreement, (0.484 for chicken, 0.554 for human and 0.542 for mouse) (Table 2). This result is consistent with the common opinion that chicken retains more ancestral adjacent gene pairs, whereas the human and mouse lineage have undergone accelerated rearrangement rates. Further, this finding also indicates that despite having occurred 450 Mya, the rearrangement distance is far from reaching saturation, which explains why the reconstruction of an ancestral vertebrate genome is reasonably effective [27], [30].

**Table 2:** Relative DCJ distance among the WGD ancestor, chicken, mouse and human.

	WGD ancestor	chicken	mouse
chicken	0.484	–	–
mouse	0.542	0.169	–
human	0.554	0.152	0.054

**Figure 7:**
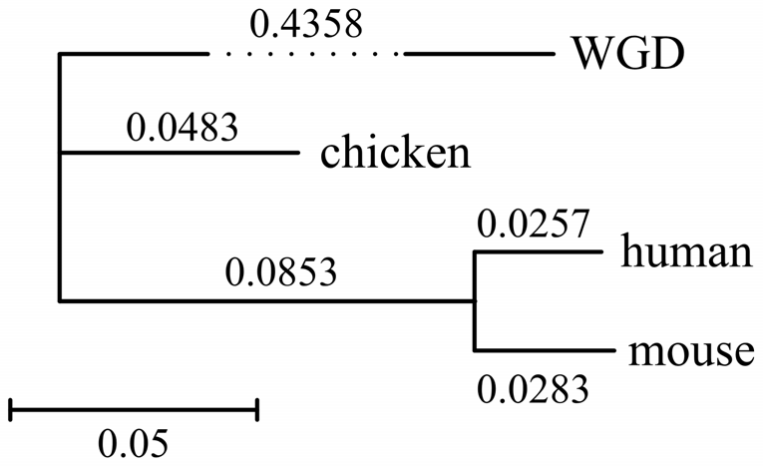
DCJ distance-based NJ tree of human, chicken, mouse and their WGD ancestor.

We also inferred relative DCJ distances for human-mouse, human-chicken and mouse-chicken (Table 2) and constructed an NJ tree with inferred distances (Figure 7). Considering that the divergence time of chicken-mammal is approximately 300 My, and the 2R–WGD occurred approximately 450 Mya, the rearrangement rates before and after chicken–mammal common ancestor are 0.4358/150 = 0.00296 DCJ per million year per gene, and (0.0853+0.0483+ (0.0257+0.0283)/2)/(300×2) = 0.000268 DCJ per million year per gene, respectively. This indicates that the rearrangement rate tends to decelerate later in evolution following the 2R-WGD.

## Discussion

In this study, we present a new heuristic algorithm for genome aliquoting. Overall, the algorithm has greater accuracy than earlier algorithms developed for this purpose, especially when the simulated distance is high. When we applied the algorithm to vertebrate genomes, the results suggested that the rearrangement rates of vertebrate genomes may decelerate later in evolution after the 2R–WGD. A more conclusive result requires the inclusion of more genes in the analysis, which is not possible with the current version of algorithm due to its inability to handle gene losses. Nevertheless, the accuracy of this algorithm can be further improved in several aspects at the cost of increasing running time (such as increasing the parameter **Depth**), which can be highly useful for situations wherein running time is not a priority. Additionally, the ideas of this heuristic algorithm may also be extended to other rearrangement problems, such as the genome median problem, with respect to DCJ operation, which is NP–hard.
